# Oxygen Sensing: Physiology and Pathophysiology

**DOI:** 10.3390/antiox11051018

**Published:** 2022-05-21

**Authors:** Philip I. Aaronson, Asuncion Rocher

**Affiliations:** 1 Department of Inflammation Biology, School of Immunology and Microbial Sciences, Faculty of Life Sciences and Medicine, King’s College London, London SE1 1UL, UK; 2 Departamento de Bioquímica, Biología Molecular y Fisiología, Facultad de Medicina, University of Valladolid, 47005 Valladolid, Spain; 3 Unidad de Excelencia Instituto de Biología y Genética Molecular (IBGM), University of Valladolid-CSIC, 47005 Valladolid, Spain

Oxygen is such an essential element for life that multiple mechanisms have evolved to maintain oxygen homeostasis, including those which detect decreases in arterial O_2_ and generate adaptive responses to hypoxia. In mammals, oxygen sensing mechanisms are found in erythropoietin-producing cells, peripheral chemoreceptor cells (carotid and aortic bodies), pulmonary artery smooth muscle cells, pulmonary neuroepithelial cells, chromaffin cells, and some specific types of neurons.

Short-term responses to hypoxia mainly include chemoreceptor-mediated reflex ventilatory and hemodynamic adaptations to low oxygen whereas prolonged exposure to hypoxia can elicit sustained physiological responses including the transition from aerobic to anaerobic metabolism and enhancement of oxygen-carrying capacity in the blood. Cellular adaptation to hypoxia is mediated largely by an oxygen-sensitive transcription factor, HIF-1, which can induce up-regulation of different genes, including those for redox status-controlling enzymes. These cope with the cellular effects related to decreased oxygen levels, but their overactivation can trigger pathological effects.

Knowledge of the pathophysiology of oxygen sensing is highlighting the medical relevance of carotid body (CB) dysfunction and its impact on disease mechanisms, including sleep apnea, hypertension, and heart failure. Results from different laboratories support an essential role of oxidative stress in the generation of CB dysfunction and in the progression of cardiorespiratory and metabolic disorders induced by chronic hypoxia, mainly when it occurs intermittently.

This Special Issue brings to the reader current knowledge on the effects of hypoxia at the cellular and molecular level on several particularly sensitive organs, in a variety of physiological and pathological situations.

The carotid body (CB) is the main peripheral chemoreceptor that orchestrates the body’s homeostatic response to hypoxia. Here, CB Type 1 (glomus) cells respond to a fall in blood pO_2_ by releasing multiple neurotransmitters, thereby activating afferent nerve fibers in the carotid sinus nerve (CSN) which signal to the medulla to initiate the body’s response. The cellular mechanisms by which CB Type 1 cells sense hypoxia are controversial, but a leading proposal, based on studies in mice, is that hypoxia increases mitochondrial ROS (mitoROS) production at complex 1, an effect which is proposed to be due to reverse electron flow resulting from increased succinate oxidation by complex 2 [1]. The resulting increase in [ROS] inhibits the opening of plasmalemmal K^+^ channels, causing membrane depolarization and Ca^2+^ influx which triggers neurotransmitter release. However, there is also evidence opposing the involvement of succinate and complex 2 in O_2_ sensing by CB Type 1 cells [2], and the applicability of this mechanism to other species is undefined. This led Swiderska et al. to carry out a study, reported in this Special Issue [3], which examined the role of mitoROS, Complex 2 and succinate in CB O_2_ sensing in rats.

The authors used a combined physiological/pharmacological approach, applying hypoxia to an isolated rat CB/CSN nerve preparation in the presence and absence of various agents which modulate mitoROS generation and succinate metabolism while recording nerve firing. They also used whole body plethysmography to characterize the respiratory response of rats to hypoxia in vivo under control conditions and after IV injection of MitoT, a mitochondrial matrix antioxidant.

The key findings of the study were that both antioxidants targeting the mitochondria, and the block of complex 2-mediated succinate oxidation by dimethyl malonate, depressed the response to hypoxia in vitro. A similar effect, which manifested as an attenuation of the hypoxia-induced increase in respiratory frequency, was seen in the whole animal with antioxidant injection. However, a very substantial (≥50%) component of the response to hypoxia was resistant to all of the interventions designed to decrease mitoROS production, leading the authors to conclude that complex 2- and mitoROS-independent mechanisms also contribute importantly to O_2_ sensing by the CB in rats.

Another proposed mechanism for O_2_ sensing in the CB and elsewhere involves the metabolism of the gasotransmitter hydrogen sulfide (H_2_S). This concept was first introduced in 2006 by Kenneth Olson [4], who has written an authoritative review on this subject in this Special Issue [5]. In it, he provides a detailed description of the synthesis and metabolism of H_2_S, guides the reader through some of the complexities of its chemistry and interactions with other signal transduction pathways and cellular effector systems, and summarizes the evidence supporting its roles in O_2_ sensing throughout the body [5].

According to the model described in the review, hypoxia causes an increase in cellular [H_2_S], which then initiates the cellular response by causing the persulfidation of reactive thiols on regulatory proteins. Cellular [H_2_S] increases in response to hypoxia because its metabolism, which requires oxygen, falls in parallel with the cellular O_2_ concentration, whereas the activities of the enzymes which mediate its synthesis from cysteine and methionine are not directly regulated by the pO_2_, although in CB cells there is evidence that hypoxia can also indirectly increase H_2_S production through carbon monoxide- and protein kinase G regulated phosphorylation of cystathionine g-lyase [6]. Thus, the concentrations of H_2_S and O_2_ in cells vary inversely, such that, for example, CB Type 1 cell H_2_S release and CB-induced activity of the sinus nerve show a very similar dependence on pO_2_ over the range relevant to physiological hypoxia [7]. Moreover, evidence for the involvement of H_2_S in the regulation of vascular tone by O_2_ comes from experiments demonstrating a remarkable correspondence between the effects of hypoxia and the application of exogenous H_2_S in a wide range of different arteries from numerous species [8]. The review ends with a discussion of the relationships between reactive sulfur species (RSS) and ROS and makes two important points. One is that in vitro studies of O_2_ sensing are routinely carried out under conditions that are hyperoxic compared to those in vivo, in which case cellular RSS and ROS levels will be artifactually diminished and enhanced, respectively. The other is that certain indicators used to measure ROS are actually more sensitive to RSS. Both considerations suggest that the role of H_2_S in O_2_ sensing has been underestimated and needs to be examined more carefully.

Pulmonary hypertension is classified into five classes, the first of which is generally referred to as pulmonary arterial hypertension (PAH). Notably, PAH occurs preponderantly in females, who however have a better prognosis than males. It’s thought that higher levels of estrogen in females are in part responsible for both of these aspects of PAH, but as explained in a timely review of this subject by Kostyunina and McLaughlin [9], genes on the X and Y chromosomes also play an important role in this sexual dimorphism.

As described in their paper, the use of the Four Core Genotypes and XY* mouse models, which enable the influence of the sex chromosomes to be distinguished from that of sex hormones, suggest that, at least in mice, the sex-determining region of the Y chromosome (STY) confers protection against the development of PH. In agreement with this concept, the presence of the STY in cultured cells has been shown to be associated with a higher expression of the BMPR2 receptor [10], the downregulation of which causes a profound increase in the risk of developing PAH.

Although most of the approximately 2600 genes on the X chromosome are equally expressed in males and females because one X chromosome in females is inactivated, incomplete inactivation or mosaicism, which can ameliorate effects of disadvantageous gene alleles in the maternally inherited X chromosome in females but not males, means that genes on the X chromosome can also contribute to sexual dimorphism. As the review describes, these encode numerous proteins believed to contribute to the development of PH, including transcription factors and important regulators of ROS production, apoptosis, inflammation, and vascular remodeling. In addition, the abnormal expression of certain miRNAs is increasingly being seen as playing key role in PH pathogenesis [11], and one of these, miR223 [12], is encoded by a gene on the X chromosome and can exhibit sexual dimorphism. The authors conclude that a better understanding of the role of the sex chromosomes in PH is likely to uncover novel targets which can be exploited to develop treatments tailored to both sexes.

The study by Morales-Cano et al. [13] reported in this Special Issue assessed important aspects of the selectivity of drugs currently of interest in the treatment of pulmonary hypertension (PH). The pulmonary vascular endothelium normally exerts a vasodilating, antiproliferative influence on pulmonary artery smooth muscle cells by releasing nitric oxide and prostacyclin. These pathways are downregulated in PH, whereas the release of vasoconstricting and pro-proliferative endothelial factors such as endothelin and 5-HT increases. This imbalance contributes to the pulmonary-specific vasoconstriction and remodeling which characterizes this condition, and every drug currently available to treat PH, including prostacyclin receptor agonists, phosphodiesterase inhibitors, endothelin antagonists and riociguat, acts to restore a more normal balance to endothelial regulation of the pulmonary vasculature [14].

However, the vasodilating properties of these drugs can have consequences that limit their usefulness. Patients with PH may have poorly ventilated lung regions in which hypoxic pulmonary vasoconstriction is restricting blood flow. This localized vasoconstriction minimizes V-Q mismatch, which can therefore be worsened by dilation of pulmonary arteries in hypoxic areas of the lung [15]. Ideally, therefore, vasodilators used to treat PH would be less potent under hypoxic conditions. It is also preferable that these drugs act selectively on pulmonary vs. systemic arteries to avoid side effects such as hypotension and oedema.

Morales-Cano and colleagues [13] used small vessel myography in isolated arterial rings from humans and rats to determine the extent to which more than twenty different vasodilating drugs, including treprostinil, sildenafil and riociguat which are currently used to treat PH, and others which target various contractile pathways of potential interest in managing this condition, demonstrate selectively for pulmonary vs. systemic arteries and under normoxic vs. hypoxic conditions. Their key finding is that none of the drugs they tested showed either type of desirable selectivity for vasodilation; in fact, some of the drugs were less efficacious in pulmonary arteries under normoxic compared to hypoxic conditions. The authors conclude that successful treatment for types of PH which are associated with pulmonary hypoxia, the need for which is currently unmet, will require more consideration to be given to their selectivity for pulmonary arteries under normoxic conditions.

Physiomimetic experimental models are quickly evolving thanks to the advances in organ-on-a-chip and tissue engineering technologies. The review from Otero et al. [16] in this Special Issue discusses the importance of developing physiomimetic approaches aiming to reproduce healthy and pathological cell environments in model systems, in part by creating a more physiologically accurate gradient of oxygenation within multicellular preparations than those achieved using traditional in vitro approaches. Alongside the explosion of these novel experimental approaches, different bioengineering techniques have been developed and improved. Increased affordability and popularization of 3D bioprinting, fabrication of custom-made lab-on-a chips, development of organoids and availability of versatile hydrogels are factors facilitating the design of tissue-specific physiomimetic in vitro models.

The control of oxygen delivery to cells is essential to reproduce physiological conditions involving hypoxia and the generation of reactive oxygen species. As the lung is the organ where gas concentration changes are of major interest, most efforts are being made to mimic the pulmonary system in physiomimetic models. Regarding other tissues and organs, O_2_ monitoring is important for tissue engineering and regenerative medicine since stem cell differentiation, cell therapy and engraftment of progenitor cells into the target tissue seem to be more efficient at specific levels of oxygen. The authors review the novel advanced 3D devices which are introducing integrated biosensors capable of monitoring oxygen consumption, pH and cell metabolism. These biosensors seem to be a promising solution to better control the oxygen delivery to cells and reproduce disease conditions involving hypoxia. Otero et colleagues [16] conclude that the development of more sophisticated bioreactors and the use of novel oxygen biosensors will provide new opportunities to better understand human physiology, improve cell therapy and regenerative medicine, and determine how oxygen dysregulation could foster the development and progression of multiple diseases.

Hypoxia induces several responses at cardiovascular, pulmonary, and metabolic levels which may lead to chronic disease. Initial responses to hypoxia are compensatory and induce activation of cardiorespiratory protective mechanisms. However, whenever hypoxia is prolonged, the chronic activation of cellular responses induces sustained modifications that, as well as resulting in acclimatization, may produce maladaptive changes which increase cardiovascular risk. Permanent high-altitude inhabitants have augmented risk and prevalence of chronic hypoxic PH, right ventricular hypertrophy, and cardiopulmonary remodeling. Similar responses are seen in adults exposed to chronic intermittent hypoxia (CIH), as seen in obstructive sleep apnea (OSA), the most common sleep-related breathing disorder [17]. OSA is characterized by repetitive episodes of apneas (airflow cessation) or hypopneas (airflow reduction) due to partial or complete collapse of the upper airway during sleep. Intermittent hypoxia in OSA has been recognized as being responsible for most OSA-related comorbidities, particularly systemic hypertension (HTN). Currently, understanding of the pathophysiological mechanisms linking CIH and cardiovascular function is limited by the diversity of hypoxic phenomena in human subjects and the multiple comorbidities, including obesity, insulin resistance and previous cardiac alterations [17].

Animal models allow researchers to evaluate the pathophysiology of OSA and explore different treatments. The most widely used and best described in the literature for sleep-disordered breathing is the intermittent hypoxia (IH) model, which simulates the most important aspects of OSA. In this model, small animals, such as mice and rats, are placed in specific chambers in which environmental gases are tightly controlled.

The influence of intermittent hypoxia in pathological situations is detailed in three further papers by Martins et al. [18], Correia et al. [19]., and Prieto-Lloret et al. [21] which provide new insights into the mechanisms of metabolic dysfunction, maladaptive mechanisms in the renal cortex and pulmonary hypertension in a rat model subjected to CIH, paving the way to new research questions, therapy options and precision medicine for OSA.

Several studies have shown a link between OSA and the development of insulin resistance; however, the main event that triggers insulin resistance in OSA remains to be clarified. The article by Martins et al. [18] investigates the effect of mild and severe CIH on rat whole-body metabolic dysregulation and on visceral adipose tissue dysfunction as well as on the contribution of obesity to CIH-induced dysmetabolic states and tissue dysfunction, focusing on hypoxia, angiogenesis, inflammation, oxidative stress, and adipose tissue metabolic function. The authors provide mechanistic insights demonstrating that, although both mild and severe CIH lead to whole-body insulin resistance and hyperinsulinemia, these early dysmetabolic states are not associated with increased weight gain, adipocyte perimeter, adipose tissue hypoxia, angiogenesis, or oxidative stress.

They also demonstrate that obesity-induced adipose tissue dysfunction is associated with increased hypoxia and decreased angiogenesis, together with increased oxidative stress in the adipose tissue and alterations in its metabolism but does not affect inflammatory markers. Interestingly, CIH in obesity does not aggravate metabolic dysfunction and attenuates obesity-induced adipose tissue dysfunction. The reduction in adipocytes perimeter found in obese animals exposed to CIH suggests a higher metabolism of the adipose tissue and/or increased energy expenditure that might be responsible for the reduction in body weight. Altogether, their results suggest a protective role for CIH against some factors that usually contribute to adipose tissue dysfunction in obesity, mainly tissue hypoxia and inflammation, as postulated by other authors [18].

Martins and colleagues [18] conclude that adipose tissue dysfunction is not the main trigger for early-stage insulin resistance in CIH and that CIH at this stage might protect against the deleterious effects of obesity in adipose tissue function.

OSA and hypertension promote functional and structural kidney damage and increases oxidative stress and the activities of the renin-angiotensin-aldosterone and sympathetic nervous systems [17]. There are, however, few studies dedicated to the impact of CIH on kidney molecular signatures and these have produced conflicting data, possibly due to the variable frequency, intensity, and duration of CIH paradigms used. The study by Correia et al. [19] in this Special Issue adds novel players for the molecular mechanisms of CIH-HTN and contributes to a better understanding of the phenotype of OSA-HTN, in line with the major current challenges in OSA diagnostic and management strategies.

Correia and colleagues [19] hypothesize that an interplay between the aryl hydrocarbon receptor (AhR) and the cysteine-related thiolome in the kidney cortex underlies mechanisms of (mal)adaptation to chronic intermittent hypoxia (CIH) that promote arterial hypertension (HTN). The aryl hydrocarbon receptor (AhR) is a ligand-activated transcription factor belonging to the basic helix-loop-helix/Per-Arnt-Sim (bHLH/PAS) family. It is implicated in diverse and relevant physiological processes such as control of cell cycle, immune, renal, and cardiovascular system development, and oxidative balance [20]. In the canonical pathway, ligand binding evokes AhR translocation to the nucleus, where its dimerization with the AhR nuclear translocator (ARNT) promotes the transactivation of target genes, namely CYP1A1, that represent the hallmark of AhR activation [20]. AhRs may also interact with other pathways, some of which are relevant in the context of oxidative homeostasis, the generation of reactive oxygen species (ROS) and regulation of the expression and activation of antioxidant genes, including superoxide dismutase 1 (SOD-1).

Here, the authors investigate the impact of CIH on the Cyp1a1 protein level and cysteine-related thiol pools using a rat model of CIH-HTN and measuring these endpoints after exposure to short-term, mid-term (pre-HTN) and long-term IH (established HTN). Their results suggest that acute and chronic intermittent hypoxia impact the interaction between AhR-CYP1A1 and cysteine-related thiolome, causing opposite effects on Cyp1a1 and the thiolome. While short-term IH decreases Cyp1a1 and increases protein-S-thiolation and total GSH, long-term IH increased Cyp1a1 expression and decreased protein S-cysteinylation and GSH. Moreover, free oxidized cysteine increased with the same temporal pattern as AhR activation. Data suggest that increased S-thiolation of proteins might represent a protective mechanism for CIH impact on blood pressure and may provide the basis for innovative antioxidant mechanisms of action. The different impact of short- and long-term IH on cysteine-related thiolome highlights the importance of the use of different CIH paradigms in pharmacological studies of antioxidants. This study supports Cyp1a1 as a biomarker of OSA severity and oxidized pools of cysteine as risk indicators of OSA-HTN.

As previously mentioned, data from different studies implicate OSA in the development of hypertension, cardiac ischemia, congestive heart failure, arrhythmias, cerebrovascular disease, and stroke [17]. In turn, there is growing evidence that CIH may also compromise the pulmonary circulation, causing PH in both OSA patients and animal models. In this Special Issue, Prieto-Lloret et al. [21] compare hemodynamics, vascular contractility, and L-arginine-NO metabolism in two rat models of PH, one associated with chronic sustained hypoxia (CSH) and the other with CIH exposure. They demonstrate that, while CSH and CIH cause several common effects, such as increased hematocrit, weight loss, and increased pulmonary arterial pressure (PAP), CSH appears to have a greater effect on the pulmonary circulation, whereas the effects of CIH are apparently more directed to the systemic circulation. Furthermore, their results suggest that the endothelial dysfunction evident in pulmonary arteries with both hypoxia protocols is not due to an increase in methylated arginines in these arteries, although an increase in plasma SDMA could contribute to the apparent loss of basal NO-dependent vasodilation and thus to the increase in PAP that results from CIH [21].

During the process of writing this editorial, the most senior of our authors in this Special Issue, Prof. Robert Fitzgerald passed away on 20 April at the age of 90. We are privileged to have been able to capture his unique insights into the physiology and pathophysiology of oxygen sensitivity in his latest work in *Antioxidants* [22].

Prof. Fitzgerald was a pioneering researcher in respiratory physiology and a longstanding member of the faculty at the Johns Hopkins University School of Medicine. He received his PhD in physiology from The University of Chicago and did post-doctoral training at UCSF/CVRI in San Francisco and in France at Université de Nancy and Université de Paris. Returning to his faculty position at The Johns Hopkins Medical Institutions in 1967, he eventually became Associate Chair, then Acting Chair of the Department of Environmental Health Sciences in the Bloomberg School of Public Health with joint appointments in the Departments of Physiology and of Medicine in the School of Medicine.

Prof. Fitzgerald’s research career focused on cardiopulmonary physiology, especially cardiopulmonary control. He was a pioneer and world-renowned expert in carotid body function, especially when it came to establishing the hypercapnic sensitivity of the carotid body and the cholinergic hypothesis of chemotransduction. His CV includes over 200 contributions to peer-reviewed publications, and he was elected a Fellow of the Royal Academy of Medicine of Ireland in 1990.

Prof. Fitzgerald was one of the founders of the International Society of Arterial Chemoreceptors (ISAC) in 1987, along with Carlos Eyzaguirre, Sal Fidone, Sukhamay Lahiri and Donald McDonald. He was intensely committed to the Arterial Chemoreceptor Society and its research on the carotid body and its importance in cardiovascular homeostasis right up until his death.

We offer this Special Issue as a tribute to Prof. Bob Fitzgerald and to the other eminent scientists who made vital contributions to the field of chemoreception, and particularly to our understanding of the carotid body, for which we are all grateful, and who sadly have passed away during the last decade: **Prof. Sukhamay Lahiri** (2009), **Prof. Constancio Gonzalez** (2015), **Prof. Machiko Shirahata** (2016), and **Prof. Chris Peers** (2018). We have lost them, but their legacy continues through the research of many scientific heirs, students, collaborators, and friends.
